# The emergence of meal delivery applications: a research agenda to advance the next decade of progress in nutrition

**DOI:** 10.1038/s41430-025-01597-y

**Published:** 2025-03-19

**Authors:** Si Si Jia, Rebecca Bennett, Adyya Gupta

**Affiliations:** 1 The University of Sydney, Sydney School of Nursing and Midwifery, Faculty of Medicine and Health, Sydney, NSW Australia; 2 The University of Sydney, Charles Perkins Centre, Sydney, NSW Australia; 3 Deakin University, Global Centre for Preventive Health and Nutrition (GLOBE), School of Health and Social Development, Faculty of Health, Geelong, VIC Australia

**Keywords:** Risk factors, Nutrition

## Abstract

The United Nations Decade of Nutrition was declared on 1 April 2016 to accelerate action to achieve global nutrition and diet-related non-communicable disease targets by 2025. Meal delivery applications offering takeaway meals and ready-to-eat foods from restaurant kitchen to doorstep via a third-party courier, have proliferated as a new digital dimension to traditional food environments. These digital platforms threaten to disrupt progress towards creating a health-enabling food environment. This article outlines the emergence of the digital food environment—its dimensions, actors and target users, and critically appraises the research on the public health impact of meal delivery applications to-date. We propose a research agenda to measure, monitor and mitigate the risks which meal delivery applications pose to population health and wellbeing, which may impact the United Nation’s Decade of Action on Nutrition. The rapidly evolving digital era of new technologies and innovation presents a unique window of opportunity for public health research and policy.

## Digital food environments

Digitalisation and technological innovations have transformed all aspects of life—including the way people can access food. The term ‘digital food environments’ was first conceptualised by Granheim et al. in a systematic scoping review of 357 studies [1]. The review identified online grocery retail and online food delivery services of fast food and other ready meals, as part of the dominant typology of food vendors in the digital food environment [1]. A narrative review has further characterised the digital food environment into three main components including: (1) meal delivery applications ‘apps’ (or online food delivery of fast food and other ready meals), (2) online groceries and (3) meal-kit subscription services [2]. These online platforms have increased access to unhealthy foods [3].

## Meal delivery applications

Meal delivery apps coordinate orders between customers and food retailers. High smartphone penetration, the growth of e-commerce, the rise of technology companies such as Uber, and the sharing economy have allowed meal delivery apps to flourish over the past decade [4]. The meal delivery market worldwide is projected to reach a revenue of US$435.30 billion in 2024, which reflects a tremendous increase from US$109.30 billion generated in 2017 [5]. By 2029, the number of meal delivery app users is expected to reach 2.7 billion worldwide [5]. The following are leading meal delivery apps in their respective markets across the globe [6]:

- China: Ele.me, Meituan, Dianping
- Australia and New Zealand: UberEats*, Menulog**, DoorDash
- North America: GrubHub, UberEats, Postmates*
- Europe: Gorillas, Deliveroo, Flink, Just Eat**, UberEats, Wolt
- Southeast Asia: Grab, Gojek, Delivery Hero
- South America: Rappi, iFood
- India: Zomato
  *Postmates is a subsidiary of UberEats.
  **Menulog is a subsidiary of the global Just Eat Takeaway.com network.

A growing body of evidence indicates that these applications predominantly offer unhealthy fast foods [7] which are heavily promoted through value bundles and have increased visibility on the platform [8, 9]. While the research to-date has largely been conducted in high-income countries [10], evidence indicates that the emergence of meal delivery applications (‘apps’) may exacerbate unhealthy diets contributing to non-communicable diseases [11, 12], and undermine global efforts to improve population diets and nutrition. In this article, we outline how meal delivery apps act as digital disruptors to the efforts undertaken to achieve the desired targets as outlined in the Nutrition Decade.

## United Nations Decade of Action on Nutrition

The United Nations General Assembly proclaimed the Decade of Action on Nutrition in 2016 to achieve global nutrition and diet-related non-communicable disease (NCD) targets by 2025 [13]. Creating safe and supportive environments for nutrition at all ages is one of six key action areas of the Nutrition Decade which aims to promote availability, affordability, promotion and quality of food supporting healthy diets.

In a mid-term review of the Nutrition Decade, priority actions to achieve s*afe and supportive environments for nutrition* were identified for 2021–2025. This included: first, to scale-up implementation of regulatory policies by leveraging recent momentum and learnings from country experiences such as sugar-sweetened beverage taxes, marketing restrictions and front-of-pack nutrition labelling [14]. Second, to develop and implement regulatory or voluntary approaches to increase availability of healthy choices and create a supportive environment for nutrition [14]. Third, to revitalise action in key policy areas which have stagnated or deteriorated recently [14].

**Fig. 1:**
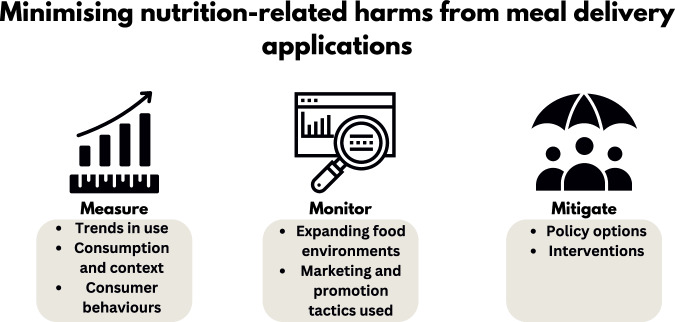
Overview of the proposed research agenda to measure, monitor and mitigate the nutrition-related harms which meal delivery apps pose.

The digitalisation of food environments and emergence of meal delivery applications have likely undermined efforts towards these priority actions which include creating safe and supportive environments for nutrition. We outline a research agenda to measure, monitor and mitigate the risks which meal delivery apps pose to population diets and health. This agenda aims to highlight meal delivery apps as a disruptor to the Nutrition Decade and advance actions on improving global nutrition into the next decade and beyond (Fig. 1).

## Research agenda

### Measuring the public health impact of meal delivery apps

It is essential for future research to measure the extent to which the meal delivery apps impact health and identify the risks which meal delivery apps pose to current food environments. This includes measuring what products are being sold and how, how and why consumers use meal delivery apps and the trends of its use across different time periods (when) and population groups (by whom).

In most countries globally, the popularity of meal delivery apps is rising rapidly, primarily due to the easy access and the convenience of ordering from a wide variety of food retailers with home delivery options [15, 16]. This has led to an increased use of, and dependence on, meal delivery apps for food purchases and consumption of food. A systematic review has also shown that meal delivery app use increased during COVID-19 restrictions when physical access to foods was limited [17]. Since the COVID-19 pandemic, these platforms are swiftly expanding their services to now include groceries, alcohol and other essentials (e.g., pharmacies), making them more lucrative for consumers. Moving forward, it is imperative to observe whether meal delivery apps continue to increase in usage or popularity, in addition to any changes in products (such as food, alcohol) or food outlets available over time.

Research has established that meal delivery apps offer an abundance of energy-dense and nutrient poor foods [18]. Moreover, a systematic scoping review suggests that meal delivery apps appear to have a negative impact on health by promoting the sale and consumption of unhealthy foods and beverages [19]. Despite these findings which suggest high availability and promotion of unhealthy foods, there is limited research on what users of meal delivery apps are purchasing and consuming, and how their intake of these foods, may be contributing to poorer health outcomes. Of concern, market research from Australia suggests that young people aged between 16 to 35 years are the highest users [20]. This is a critical life stage where many lifestyle behaviours are formed [21] which are known to be notoriously difficult to undo or change in later adulthood [22]. This is also the life stage when prevalence of overweight and obesity increases the fastest [23]. Adolescents especially, are a target market for food and beverage companies as they are viewed as ‘lifelong’ customers to build brand recognition, brand preference and brand loyalty [24]. It is therefore critical to continue measuring how young people are using meal delivery apps.

A study using mobile ecological momentary assessment, captured 124 meal delivery events from 58 unique participants between 16–35 years and showed majority of orders were unhealthy foods such as pizza and fried chicken [25]. Further research is needed to measure the use of meal delivery apps in real-time, and how meal delivery apps contribute to an individual’s overall diet or used as a source of everyday food. Future studies could consider establishing large ‘digital cohorts’ to capture online food marketing data used by meal delivery apps targeting young people in near real time. These digital cohorts are currently being discussed as a data collection method in studies on diabetes [26] and e-cigarette use [27].

### Monitoring the reach and public health impact of meal delivery apps

A vast amount of research on ‘neighbourhood food environments’ have typically focused on investigating associations between stores or restaurants within a geographic boundary such as postcodes or census tracts [28]. The evidence surrounding the influence of local food environments on health are mixed. A systematic review of 38 studies conducted by Caspi et al. in 2012, showed moderate evidence to support the hypothesis that neighbourhood food environments influence dietary health [29]. Many included studies reported an association between food accessibility and dietary outcomes such as a higher vegetable consumption among those that live furthest from a fast-food outlet. Despite this, several other studies reported a null association [29]. These inconsistent findings are reported widely across additional systematic reviews in the field [30–32].

Emerging research suggests that meal delivery apps allow users to order food from over 3 km away [18] and this could alter any existing associations between food environments and dietary outcomes. The expansive nature of meal delivery apps may change the concept of ‘neighbourhood’ or ‘local food environments’, which is typically comprised of food outlets within a 1 km radius of a person’s home [33]. Meal delivery apps can now provide individuals access to a much wider range of outlets [7, 18]. Therefore, individuals and communities may now be experiencing ‘hybrid’ food environments with access to foods within their local environments, in addition to their online food environments [34]. Hybrid food environments may exacerbate local food environments by increasing access to all types of foods, including unhealthy foods. It is unclear how this may impact vulnerable groups of lower socioeconomic status, as research suggests there is a higher density of fast-food outlets in these deprived areas [35, 36]. Monitoring the reach and impact of meal delivery apps on local food environments is therefore a key research priority. The World Health Organization Regional Office for Europe and research groups in Australia have developed monitoring tools and dashboards [34, 37]. These have capacity for further surveillance of the meal delivery application market, sales volumes and customer profiles.

Marketing techniques used by meal delivery apps have also been highlighted as a concern that requires further monitoring. There is some evidence to suggest these have been employed to primarily promote energy-dense nutrient-poor foods on meal delivery apps [7]. However, there is no evidence on the extent to which the consumers are exposed to and engaged with the marketing techniques on meal delivery apps. The mechanism through which the marketing techniques influence purchase and consumption of foods on meal delivery apps also remains unclear. These are important areas for further monitoring which can be used to urge government action on the regulation of meal delivery apps.

### Mitigating the public health risks associated with meal delivery apps

Currently, meal delivery apps operate with little government oversight [10]. This is despite calls from public health for greater regulation in this sector [38, 39] and demonstrates an important research gap needed to mitigate the public health risks associated with meal delivery apps. Research is needed to ascertain which existing policies do, or do not address meal delivery apps and to what extent. The novel, dynamic nature of these apps means that regulations targeting them will also need to be innovative, and adaptable as the platforms evolve and change over time. In response, research will need to inform the development and evaluation of new and existing regulations for their effectiveness in limiting the public health impact of meal delivery apps.

Importantly, while use of meal delivery apps is on the rise, access to the other traditional modes of food purchasing continue to remain relevant [16]. This indicates an opportunity to strengthen the existing regulations for traditional food environments affecting food served in restaurants or cafes. These policies may be adapted to include meal delivery apps and apply to online food environments. For example, ensuring existing kilojoule menu labelling laws are applied to the same standard online compared to physical stores could be a way to inform purchasing decisions [40]. Additionally, there have also been suggestions to enhance the positioning of healthy options in the app’s menu, through algorithmic boosting of healthier options [38]. However, these initiatives need to be evaluated for their impact on food purchasing decisions.

Research analysing the corporate strategies and activities of meal delivery apps will be important to understand what activities they may use to attempt to resist government regulation and help to inform public health messaging to hold this sector accountable. Previous studies have analysed commercial activities of the food industry and found similarities in strategies and tactics used by food, alcohol and gambling industries [41]. This may provide insights into how meal delivery apps may also use their corporate power to influence the policy making process. Understanding these tactics can help inform the development of potential innovative solutions to minimise consumers’ exposure and their impact on consumers’ food purchasing behaviours [41].

### Conclusion

This research agenda highlights the importance of addressing meal delivery apps as digital disruptors to safe and supportive environments for nutrition. Future research areas outlined in this article include measuring, monitoring and mitigating against risks which meal delivery apps pose to population diets and nutrition. As the end of the Nutrition Decade is approaching, it is critical to maintain the momentum of actions to improve safe and supportive environments for nutrition and advance the global nutrition agenda into the next decade.
